# Smoking and drinking behavior, knowledge, and attitudes among urban and rural public-school students in Efate Island, Vanuatu: a comparative study

**DOI:** 10.1186/s13690-022-00929-9

**Published:** 2022-07-18

**Authors:** Emi Nakaseko, Sayaka Kotera, Minato Nakazawa

**Affiliations:** 1 Department of Nursing, Faculty of Health Sciences, Kansai University of International Studies, 18-1 Aoyama, Shijimi-Cho, Miki-City, Hyogo, 673-0521 Japan; 2 Department of Public Health, Graduate School of Health Sciences, Kobe University, 7-10-2 Tomogaoka, Suma-ku, Kobe-City, Hyogo 654-0142 Japan

**Keywords:** Tobacco smoking, Alcohol drinking, Students, Rural–urban, Vanuatu

## Abstract

**Background:**

Underage smoking and drinking are public health issues in Vanuatu. This study aims to describe the behavior, knowledge, attitudes, and perceptions of parents, siblings, and peers regarding smoking and drinking among urban and rural public-school students in Vanuatu.

**Methods:**

This cross-sectional study included 358 students (urban, 217; rural, 141; aged 12–14 years) from the public schools in Efate Island, Vanuatu. Data were collected using self-administered questionnaires. Chi-square and Mann–Whitney U tests were used to determine the rural–urban differences.

**Results:**

Urban students showed a higher prevalence of ever smoking (13.5%), ever drinking (16.9%), intention to smoke (11.1%), and intention to drink (14.0%) compared to rural students (10.3%, 8.3%, 5.8%, and 9.5%, respectively); although a significant difference was only observed in the prevalence of ever drinking. Urban students were more likely to be aware of the health hazards of substance use and showed higher self-efficacy to refuse tobacco and alcohol compared to rural students. Parents in rural areas were less likely to talk about the health hazards of substance use with their children and were more likely to offer tobacco or alcohol to them compared to parents in urban areas.

**Conclusions:**

The results provide evidence of rural–urban differences in the behavior, attitude, knowledge, and perceptions of parental behavior regarding smoking and drinking. The findings suggest that issues related to underage smoking and drinking differ between urban and rural students. Future intervention programs for reducing underage smoking and drinking should be adapted in recognition of urban and rural differences.

## Background

Tobacco and alcohol use have been recognized as major risk factors for non-communicable diseases (NCDs) [1]. Adolescence is a critical risk period for the initiation of smoking, drinking, and illicit substance use [2]. Smoking by youth has immediate adverse health consequences, including addiction, and accelerates the development of chronic diseases over the full life course [3]. As for alcohol use, drinking by teenagers increases the risk of becoming a heavy drinker and developing an alcohol disorder as young adults [4]. In addition, alcohol abuse is associated with road traffic injuries, interpersonal violence, and falls. Notably, alcohol consumption is responsible for 17.6% of all injury deaths globally in 2016 [5]. As a result, prevention of underage smoking and drinking, among many other factors, is important to reduce the NCD health burden.

Low- and middle-income countries (LMICs) have been demonstrated to have the highest burden of tobacco- and alcohol-related diseases in the world [1, 5] and have shown corresponding high rates of adolescent smoking and drinking [6, 7]. Studies have already demonstrated the need for interventions to reduce tobacco and alcohol use in young adolescents in LMICs [6, 7]. Furthermore, NCDs are an important driver of premature (age < 70 years) deaths in the majority of the Pacific Island countries (PICs), with measurably higher mortality rates than other LMIC global averages [8]. The Republic of Vanuatu, a PIC, was classified as an LMIC by the World Bank [9], and faces an increasing burden of diseases associated with tobacco and alcohol use. In fact, tobacco use accounts for 17.8% of deaths among men and 7.3% among women; both percentages are higher than the average death rates among men and women in countries classified as medium by the Human Development Index (HDI) [10]. Additionally, in Vanuatu, alcohol accounts for 20.2% and 14.5% of liver cirrhosis cases among men and women, respectively [5].

Vanuatu suffers from the health concerns of underage smoking and drinking. The Global School-based Student Health Survey (GSHS) involving students aged 13–15 years showed that the prevalence of current smokers was 19.4% and 27.5% in males and 8.2% and 14.9% in females, for 2011 and 2016, respectively; meanwhile, the prevalence of current drinkers was 10.3% and 15.4% in males and 5.8% and 9.7% in females, respectively [11, 12].Importantly, the prevalence of current smokers and drinkers among–students aged 13–15 years increased substantially from 2011 to 2016 for both sexes. Further, another study showed that more males and females aged 10–14 years continued to use tobacco daily on average in Vanuatu compared to other medium-HDI countries [10]. Vanuatu ratified the WHO’s Framework on Tobacco Control (FCTC) in 2005. Moreover, in 2008, it enforced the Tobacco Control Act [13], which prohibits the sale of tobacco products to individuals below 18 years. Further, the Liquor Licensing Act in Vanuatu prohibits the sale of liquor to individuals below 18 years and alcohol consumption by individuals below 18 years in Vanuatu [14]. The Vanuatu NCD Policy and Strategic Plan 2016–2020 was introduced to raise awareness about the health hazards of tobacco and alcohol use among students [15]. However, effective prevention programs for school-aged students may not have progressed sufficiently. Unless appropriate measures are taken, the prevalence of underage smoking and drinking will continue to increase, which, in turn, may contribute to premature NCD deaths in Vanuatu.

Many previous studies in upper middle- and high-income countries (UMICs) [16–18] and LMICs [19, 20] indicated that there were urban–rural differences in smoking and drinking behaviors and attitudes among teenagers due to differences in socioeconomic and environmental factors. Thus, it is posited that there are differences in smoking and drinking behaviors, attitudes, and knowledge among urban and rural teenagers in Vanuatu. A previous study [21], which examined the lifestyles of sixth to eighth grade students showed that the percentage of ever drinkers was significantly higher among urban students than rural students, as was the level of health knowledge of NCDs, favorable health attitudes, and the availability of parental health guidance. However, because this previous study did not focus on smoking and drinking, it did not provide an adequate account of smoking- and drinking-related knowledge and attitudes among teenagers in Vanuatu. Furthermore, many studies indicated that teenagers’ smoking and drinking behaviors or attitudes were influenced by smoking and drinking behaviors or attitudes of their parents, siblings, and peers [22–25]. However, few studies have investigated the smoking and drinking behaviors or attitude of student’s parents, siblings, and peers in PICs, including Vanuatu. Underage smoking and drinking might contribute to a country-wide rise in NCDs. Therefore, it is necessary to determine baseline data on smoking- and drinking-related behavior, knowledge, and attitudes among teenagers from urban and rural areas in Vanuatu to establish a more effective intervention program aimed at reducing underage drinking and smoking. This study aims to describe students’ behavior, knowledge, and attitudes regarding smoking and drinking, in addition to their perceptions of parental, sibling, and peer behavior with regard to these activities. Data are analyzed for urban and rural public-school students in Vanuatu, highlighting the rural–urban differences. The findings of this study could provide useful data for evidence-based intervention programs based on the characteristics of smoking- and drinking-related behavior, knowledge, and attitudes among teenagers from urban and rural areas in Vanuatu. The study findings may also be used as a reference for other LMICs and PICs.

## Methods

### Participants and procedures

This cross-sectional school-based study was conducted in March 2019. The target population of the study was sixth grade (aged 11 to 12 years), seventh grade (aged 12 to 13 years), and eighth grade (aged 13 to 14 years). This study was conducted in cooperation with the Japan International Cooperation Agency (JICA) Vanuatu Office, the Vanuatu Ministry of Health, the Vanuatu Ministry of Education and Training, and the Shefa Provincial Education Office. Convenience sampling was used to identify and recruit sample schools from both urban and rural areas on Efate Island, Vanuatu. The schools were also selected based on consultation with the Shefa Provincial Education Office, which is responsible for the public schools in Efate Island and the Vanuatu Ministry of Education and Training. To represent the urban areas, one school in the capital city of Port Vila was selected, and to represent the rural areas, one school located approximately 46 km from Port Vila by road was selected. Both sample schools are primary schools, called center schools, that educate students from grades 1 to 8. Although students in Vanuatu should compulsorily complete six years of primary education, the center schools provide eight years of education. The total number of the students of urban sample school was 659 and that of rural sample school was 343 in March 2019. The sample size was calculated using power analysis with G*power 3.1 software (University of Dusseldorf, Germany), assuming the following: α error prob = 0.05, power (1-βerror prob) = 0.8, effect size w = 0.3 (medium effects size of Cohen [26]), and Df = 1 [27]. This yielded a minimum sample size of 145. Before commencing the survey, written informed consent was obtained from the principals of the sample schools. The parents/guardians of the students were provided with a written explanation that asked them to grant permission for their minor-aged children to participate in the study.

Data were collected using self-administered questionnaires completed by the participants in their respective classrooms. A researcher visited each classroom to distribute and collect the questionnaires, and the researcher confirmed whether their parents/guardians agreed with their participation in the study before the questionnaire survey.

In addition, the researcher provided students with information about the purpose of the study, voluntary participation, confidentiality, and anonymity. The parental confirmation and the students’ submission of the completed questionnaire was considered as consent to participate in the survey.

In total, 358 questionnaires (217 from urban school, 141 from rural school) were distributed and completed.

### Measures

The questions on smoking and drinking behaviors and the level of parental involvement were based on the Vanuatu GSHS [28]. The questions on self-efficacy to refuse tobacco and alcohol from their peers, as well as knowledge of and attitudes toward smoking and drinking, were designed based on the core question of the Global Youth Tobacco Survey [29]. This study defines a parent as a man and/or a woman who currently lives with the student and takes care of them. They need not be the student’s biological parent.

The students were asked to provide their grade, age, gender, and parents’ working status. They were also asked whether they had ever tried or experimented with smoking and drinking. Smoking tobacco included cigarettes, leaf tobacco, and pipes, while drinking alcohol included drinking beer, wine, whiskey, and home brew. To assess the intention of smoking/drinking, they were asked whether they intend to smoke/drink at the age of 18 years or older. To assess the participants’ knowledge related to smoking and drinking, they were asked about the health hazards of tobacco smoking, alcohol drinking, second-hand smoke, and difficulty in stopping smoking.

To assess students’ attitudes toward smoking and drinking, they were asked whether they thought young smokers/drinkers have more friends, tobacco makes them study effectively, and alcohol makes them sleep well. To assess the self-efficacy of refusing tobacco and alcohol from peers, students were asked whether they would smoke/drink if one of their best friends offered tobacco/alcohol. The response was measured on a four-point scale (4 = definitely not, 3 = probably not, 2 = probably yes, and 1 = definitely yes); a higher score indicated higher self-efficacy.

The students were asked about the current smoking and drinking habits of their parents. To assess parental involvement, they were asked whether their parents understand their problems and worries, provide them with advice and guidance, and are usually open to communicate with them. To assess the availability of parental guidance on the health hazards of substance use, students were asked whether they had ever talked with their parents about the negative effects of tobacco, alcohol, and marijuana. To assess their perception of parental behavior, they were asked whether they had been offered tobacco/alcohol from parents and whether they had ever bought tobacco/alcohol at the request of their parents. To assess their perception of siblings and peers’ behavior, the students were asked about the current smoking/drinking habits of their siblings/peers and whether they had been offered tobacco and alcohol from their siblings/peers.

### Data analysis

Statistical analysis was performed using SPSS version 20 for Windows (IBM, Armonk, NY, USA). The level of significance was set at *p* < 0.05. Demographic data included students’ gender, mean age, grade, and their parents’ working status, and were calculated by residential area (rural–urban). Chi-square test for categorical variables and Mann–Whitney U tests for ordinal variables were performed to determine rural–urban differences in behavior, knowledge, attitudes, and perceptions of parental, sibling, and peer behavior regarding smoking and drinking. As the sample size was small, with a cell size of < 5, Fisher’s exact test was also used. Additionally, the differences in prevalence of ever smokers and drinkers by gender and grade were also examined.

## Results

The study included a total of 358 participants, with 217 (60.6%) from the urban area and 141 (39.4%) from the rural area (Table 1). Significant rural–urban differences were found in terms of grade, age, and working status of parents or guardian.

**Table 1:** Participant characteristics (*n* = 358)

	**Urban**		**Rural**		**All**		***p*-value**
	***n* = 217**		***n* = 141**		***n* = 358**		
	**n**	**(%)**	**n**	**(%)**	**n**	**(%)**	
**Gender**							
Male	115	(51.8)	76	(53.9)	191	(53.4)	0.87
Female	102	(48.2)	65	(46.1)	167	(46.6)	
**Grade**							
Year 6	56	(25.8)	41	(29.1)	97	(27.1)	0.014
Year 7	77	(35.5)	66	(46.8)	143	(39.9)	
Year 8	84	(38.7)	34	(24.1)	118	(33.0)	
**Age**							
Mean ± SD	12.73 ± 1.22		12.86 ± 1.17		12.45 ± 1.18		0.03
Father/male guardian had any job to earn salary	187	(87.0)	74	(53.6)	261	(72.9)	< 0.001
Mother/female guardian had any job to earn salary	164	(76.6)	64	(46.0)	228	(63.7)	< 0.001

Missing values excluded

Chi-square test was used to examine significant differences between students in urban and rural schools. Mann–Whitney U test was used for ordinal variable (mean age)

### Prevalence of ever smokers and drinkers by residence, gender, and grade

The overall prevalence of ever smokers and ever drinkers was 12.2% and 13.4%, respectively; and 13.5% and 16.9% in urban students and 10.3% and 8.3% in rural students, respectively (Table 2). Only the proportion of ever drinkers between the two groups was found to be statistically significant, and that of ever smokers was not. The prevalence of ever smokers and ever drinkers was significantly higher in eighth grade compared to sixth grade; however, the increase in prevalence was not significant by gender. The result that prevalence of ever smokers and drinkers tended to increase with higher grades was in line with the result of GSHS in Vanuatu [11, 12].

**Table 2:** Proportion of ever smokers and drinkers by residence, gender, and grade (n = 358)

	**Ever smoked tobacco**		**Ever drunk alcohol**	
	n	(%)	n	(%)
**All**	42	(12.2)	44	(13.4)
**Residence**				
Urban	28	(13.5)	33	(16.9)
Rural	14	(10.3)	11	(8.3)
	*p* = 0.37		*p* = 0.02	
**Gender**				
Male	27	(14.7)	26	(14.6)
Female	15	(9.4)	18	(12.0)
	*p* = 0.14		*p* = 0.49	
**Grade**				
Year6	7	(7.4)	8	(8.6)
Year7	7	(5.0)	9	(7.0)
Year8	28	(25.9)	27	(25.2)
	*p* < 0.001		*p* < 0.001	

Missing values excluded

Chi-square test was used to examine significant differences in students reporting substance use by residence, gender, and grade

Fisher's exact test for small sample size, with < 5 in a cell

### Knowledge, attitudes, and perceptions of parental, sibling, and peer behavior on smoking and drinking by residence

The proportion of students who intended to smoke tobacco and drink alcohol was relatively higher in urban students compared to that in rural students; however, no significant difference was found between urban and rural students (Table 3).

**Table 3:** Knowledge, attitudes, and perceptions of parental, sibling, and peer behavior on smoking and drinking (n = 358)

	**Urban**		**Rural**		**All**		***p*-value**
	*n* = 217		*n* = 141		*n* = 358		
	n	(%)	n	(%)	n	(%)	
**Intention of smoking and drinking** (those who answered "Yes")							
Will smoke tobacco after 18 years or older	24	(11.1)	8	(5.8)	32	(9.0)	0.09
Will drink alcohol after 18 years or older	30	(14.0)	13	(9.5)	43	(12.3)	0.21
**Knowledge on smoking and drinking** (those who answered "I think so")							
Smoking is bad for health	187	(86.6)	97	(70.3)	284	(80.2)	< 0.001
It is difficult to quit smoking	190	(88.8)	89	(64.5)	279	(79.3)	< 0.001
Secondhand smoking is bad for health	196	(91.2)	101	(72.7)	297	(83.9)	< 0.001
Drinking too much alcohol is bad for health	195	(90.7)	100	(71.9)	295	(83.3)	< 0.001
**Attitude toward smoking and drinking** (those who answered "I think so")							
Young smokers have more friends	170	(78.7)	75	(54.7)	245	(69.4)	< 0.001
Tobacco makes us study effectively	37	(17.2)	26	(19.0)	63	(17.9)	0.67
Young drinkers have more friends	171	(79.2)	89	(64.5)	260	(73.4)	< 0.001
Alcohol makes us sleep well	73	(34.1)	46	(33.1)	119	(33.7)	0.84
**Self-efficacy to refuse tobacco/alcohol** (Mean ± SD)							
Self-efficacy score to refuse tobacco (1–4)	3.76 ± 0.64		3.59 ± 0.60		3.69 ± 0.65		< 0.001
Self-efficacy score to refuse alcohol (1–4)	3.73 ± 0.63		3.59 ± 0.74		3.68 ± 0.68		0.03
**Parental involvement** (those who answered "Yes")							
Understanding problems and worries	88	(77.2)	63	(84.0)	151	(79.9)	0.25
Giving advice and guidance	106	(93.0)	68	(90.7)	174	(92.1)	0.56
Having open communication	105	(91.3)	65	(85.5)	170	(89.0)	0.21
**Parental smoking and drinking** (those who answered "Yes")							
Father/male guardian smokes tobacco	50	(23.4)	46	(34.8)	96	(27.7)	0.02
Mother/female guardian smokes tobacco	16	(7.4)	6	(4.4)	22	(6.2)	0.25
Father/male guardian drinks alcohol	95	(44.0)	55	(41.0)	150	(42.9)	0.59
Mother/female guardian drinks alcohol	47	(22.0)	16	(11.7)	63	(17.9)	0.01
**Parental health guidance on substance use** (those who answered "Yes")							
Talking about negative effect of smoking	145	(66.8)	44	(31.7)	189	(53.1)	< 0.001
Talking about negative effect of drinking	127	(58.5)	38	(27.3)	165	(46.3)	< 0.001
Talking about negative effect of marijuana use	119	(54.8)	31	(22.5)	150	(42.3)	< 0.001
**Parental request to buy tobacco/alcohol** (those who answered "Yes")							
Ever bought tobacco at the request of parents/guardians	67	(30.9)	49	(35.0)	116	(32.5)	0.42
Ever bought alcoholic drinks at the request of parents/guardians	20	(9.2)	21	(15.0)	41	(11.5)	0.09
**Parental offer of tobacco/alcohol** (those who answered "Yes")							
Offer of tobacco by parents/guardians	3	(1.4)	8	(5.8)	11	(3.1)	0.02
Offer of alcohol by parents/guardians	3	(1.4)	11	(8.0)	14	(3.9)	< 0.001
**Sibling smoking and drinking** (those who answered "Yes")							
Brother or sister smokes tobacco	43	(20.0)	25	(18.2)	68	(19.3)	0.685
Brother or sister drinks alcohol	50	(23.1)	31	(22.8)	81	(23.0)	0.939
**Sibling offer of tobacco/alcohol** (those who answered "Yes")							
Offer of tobacco by brother/sister	5	(2.3)	11	(7.9)	16	(4.5)	0.013
Offer of alcohol by brother/sister	9	(4.1)	14	(10.1)	23	(6.5)	0.025
**Peer smoking and drinking** (those who answered "Yes")							
Having friends who smoke tobacco	99	(46.5)	46	(37.1)	145	(43.0)	0.09
Having friends who drink alcohol	84	(39.4)	46	(37.1)	130	(38.6)	0.67
**Peer offer of tobacco and alcohol** (those who answered "Yes")							
Offer of tobacco by closest friends	19	(8.8)	14	(10.1)	33	(9.3)	0.66
Offer of alcohol by closest friends	12	(5.5)	14	(10.2)	26	(7.3)	0.10

Missing values excluded

Chi-square test was used to examine significant differences between students in urban and rural schools

Fisher's exact test for small sample size, with < 5 in a cell

Mann–Whitney U test for ordinal variables (self-efficacy score to refuse tobacco and alcohol)

The proportion of students who were aware of the negative effects for each of the four questions was significantly higher in urban students than rural students. Regarding attitudes toward smoking and drinking, urban students were more likely to agree that young people who smoke tobacco and drink alcohol had more friends than rural students. The self-efficacy score for refusing tobacco and alcohol was 3.76 ± 0.64 and 3.73 ± 0.63 in urban students and 3.59 ± 0.60 and 3.59 ± 0.74 in rural students, with significantly higher score among urban students compared to rural students.

More rural students (34.8%) reported perceived tobacco use by their father/male guardian than urban students (23.4%) (*p* = 0.20), and more urban students (22.0%) reported perceived alcohol use by their mother/female guardians than rural students (11.7%). The proportion of those who had ever spoken with their parents about the negative effects of tobacco, alcohol, and marijuana use were significantly higher among urban students (66.8%, 58.5%, and 54.8%, resp.) than among rural students (31.7%, 27.3%, and 22.5%, resp.).

A few of the students reported that they had been offered tobacco or alcohol by their parents or guardians at least once, with a significantly higher proportion among rural students (5.8% and 8.0%, resp.) compared to urban students (1.4% each). In addition to this, more rural students reported that they had ever been offered tobacco or alcohol by their siblings at least once (7.9% and 10.1%, resp.) compared to urban students (2.3% and 4.1%, resp.).

The results indicated that more than one-third of the students had smoking or drinking peers. The overall proportion of students who had ever been offered tobacco or alcohol by closest friends was 9.3% and 7.3%, respectively, with no significant difference between rural and urban students.

## Discussion

This study described the behavior, knowledge, attitudes, and perceptions associated with parental, sibling, and peer behavior regarding smoking and drinking between urban and rural public-school students in Vanuatu, highlighting evidence of rural–urban differences. The present study showed that there were rural–urban differences in the behavior, attitude, knowledge, and perceptions of parental behavior regarding smoking and drinking.

The results indicate that despite urban students being more likely to realize the health hazards of smoking and drinking, they exhibited a significantly higher prevalence of ever drinking compared to rural students. Although the difference was not significant, the prevalence of those who had ever smoked tobacco and intended to smoke tobacco or drink alcohol was relatively higher in urban students than in rural students. These results were consistent with a previous study [21], which reported that urban students showed a higher prevalence of ever drinking and a higher level of health knowledge compared to rural students. In addition, the 2009 National Population and Housing Census in Vanuatu showed that young people aged 15–19 years in urban areas were more likely to drink alcohol compared to those in rural areas [30]. Although the age group of the present study sample was younger than 15 years, our results were in line with the results of the national census in Vanuatu. It is worth noting that a greater level of health awareness or knowledge among urban students might not mitigate their drinking behavior.

The prevalence of ever drinkers was significantly higher among urban students than rural students. These results might be explained by environmental and socioeconomic influences. The Vanuatu Household Income and Expenditure Survey in 2010 showed that the average monthly household income was estimated at 97,500 Vatu in an urban household compared to 79,500 in a rural household [31]. Notably, our results indicate that significantly more students from urban schools had parents who were employed compared to students from rural schools. The price of a 350 ml can of beer and a pack of 20 cigarettes is approximately 230 Vatu and more than 700 Vatu, respectively, in Port Vila in 2019. Based on the monthly income estimates, beer and cigarettes might not be affordable for many Ni-Vanuatu (a native or inhabitant of Vanuatu); however, a higher household income might promote the purchase of cigarettes or alcoholic drinks in retail shops. There are more supermarkets and retail shops that sell alcoholic drinks and cigarettes in the capital city, Port Vila, compared to the residence of the rural respondents of the study. One study that explored the influence of proximity to alcohol and tobacco retailers on alcohol and tobacco use among adolescents revealed an increased risk for alcohol and tobacco use among respondents living closest to retailers [32]. Tobacco and alcohol retail environments and higher household income in urban areas might increase the accessibility of tobacco and alcohol, thereby increasing the risk of tobacco and alcohol use among students. The findings suggest that prevention programs for underage smoking and drinking should not only focus on enhancing health knowledge but should also take socioeconomic and environmental factors, such as income levels, characteristics of living environment, and accessibility of cigarettes and alcoholic drinks into account. Further research is necessary to identify the influence of environmental and socioeconomic factors on smoking and drinking behavior and perceived ease of access to tobacco and alcohol among students.

The results indicate that parents living in urban areas were more likely to talk about the health hazards of substance use with their children and less likely to offer tobacco and alcohol to their children compared to the parents living in rural areas. These results were consistent with a previous study which showed higher availability of parental health guidance among urban students compared to rural students [21]. The possible explanation for these rural–urban differences might be attributed to socioeconomic factors. The Vanuatu Demographic and Health Survey (VDHS 2013) showed that the percentage of participants who had completed more than a secondary education; those who could watch television, listen to the radio, and read a newspaper; and those who had appropriate health knowledge about tuberculosis was higher in urban areas than in rural areas for both men and women [33]. In this national survey, appropriate knowledge about tuberculosis was evaluated in order to identify how the people in Vanuatu deal with the disease, because tuberculosis is one of the oldest human diseases and continues to be a leading cause of death from an infectious disease in many countries [33]. Accordingly, we inferred that these data indicated the health literacy of the people in Vanuatu. Based on the national data, the rural–urban differences in parental attitudes and awareness might be associated with the literacy level and the availability of mass media. The results demonstrate that educational programs that enhance the health awareness and knowledge regarding smoking and drinking are necessary for parents living in rural areas. Notably, the results showed that more than 30% and 10% of the students had bought tobacco and alcohol at the request of parents, respectively; however, the sale of tobacco and liquor to persons under 18 years was prohibited in Vanuatu [13, 14]. Making students buy tobacco and alcohol might increase the risk of initiation of tobacco and alcohol consumption. One previous study indicated that adolescents who visited tobacco retail stores used tobacco more frequently at a later stage [34]. The findings suggest that intervention programs should target not only students, but also their parents. Parents should be aware that exposing their children to the tobacco and alcohol retail environment is linked with initiating smoking and drinking behavior.

In our study more than one-third of the students reported that their friends smoke tobacco or drink alcohol. Since the “friend” was not specifically defined in the questionnaire, it was unclear what types of friendship were included in their smoking or drinking friends, such as schoolfriends, childhood friends or older friends. Further research is necessary to clarify the relationships between students and their smoking or drinking friends. In relation to this, rural students showed lower self-efficacy for refusing tobacco and alcohol compared to urban students, while the difference was not significant; the percentage of those who had ever offered tobacco and alcohol from close friends was relatively higher in rural students than in urban students. Previous studies indicated that low self-efficacy to refuse tobacco and alcohol was associated with the initiation of tobacco and alcohol use among students [35, 36]. It can be presumed that students from rural areas are more likely to start smoking or drinking because of their low self-efficacy in refusing tobacco and alcohol. The WHO suggested that adolescents must enhance their negotiation/refusal skills to help them reject smoking and drinking invitations from friends through life skills education in the school [37]. Our findings suggest that enhancing negotiation/refusal skills to refuse tobacco and alcohol is more important for rural students. However, this skill is essential for all students in Vanuatu. Since our results showed that more than one-third of students had smoking or drinking friends, they might meet with their friend’s request to smoke tobacco or drink alcohol in future. Therefore, school-based life skills education is necessary for both urban and rural students to reduce underage smoking and drinking in Vanuatu.

### Limitations

Our study has some limitations. First, the study sample comprised subjects from only one island. Second, limitations of the sample size might have reduced the statistical power to determine a significant rural–urban difference. Hence, our findings may not be representative of Vanuatu. Third, there was a significant difference in average age and grade distribution between urban and rural students, perhaps due to selection bias. Forth, a self-report questionnaire might contribute to misunderstanding and acquiescence bias.

Despite these limitations, our study provides important insights that can be used by future intervention programs to prevent underage smoking and drinking in Vanuatu. Our findings suggest that in order to implement a more robust intervention program to help prevent smoking and drinking initiation among teenage students, rural–urban differences need to be considered.

## Conclusions

This study described the behavior, knowledge, attitudes, and perceptions of parental, sibling, and peer behavior with regard to smoking and drinking among urban and rural public-school students in Vanuatu, highlighting the rural–urban differences. The results indicate that despite urban students being more likely to be aware of the health hazards of smoking and drinking, they showed a significantly higher prevalence of ever drinkers compared to rural students. Rural students showed significantly lower self-efficacy in refusing tobacco and alcohol compared to urban students. Regarding parental attitudes, parents living in rural areas were less likely to talk about the health hazards of substance use with their children and were more likely to offer tobacco or alcohol to their children compared to the parents living in urban areas. In summary, the results provide evidence of rural–urban differences in the behavior, attitude, knowledge, and perceptions of parental behavior regarding smoking and drinking. The findings demonstrate that issues regarding underage smoking and drinking differ between urban and rural areas. Future intervention programs aimed at reducing underage smoking and drinking should be adapted in recognition of urban and rural differences.

## Data Availability

The datasets used and analyzed during the current study are available from the corresponding author on reasonable request.

## Abbreviations

NCD: Non-communicable disease
LMIC: Low and middle-income countries
PIC: Pacific Island countries
HDI: Human Development Index
GSHS: Global School-based Student Health Survey
FCTC: Framework on Tobacco Control
UMIC: Upper middle and high-income countries
JICA: Japan International Cooperation Agency
